# Oriented Growth of α-MnO_2_ Nanorods Using Natural Extracts from Grape Stems and Apple Peels

**DOI:** 10.3390/nano7050117

**Published:** 2017-05-22

**Authors:** Lina Sanchez-Botero, Adriana P. Herrera, Juan P. Hinestroza

**Affiliations:** 1 Department of Fiber Science and Apparel Design, Cornell University, Ithaca, NY 14853, USA; lms374@cornell.edu; 2 Department of Chemical Engineering, Universidad de Cartagena, Cartagena 130015, Colombia; aherrerab2@unicartagena.edu.co

**Keywords:** nanorods, manganese oxide, natural extracts, dye degradation, oriented attachment, green synthesis

## Abstract

We report on the synthesis of alpha manganese dioxide (α-MnO_2_) nanorods using natural extracts from *Vitis vinifera* grape stems and *Malus domestica* ‘Cortland’ apple peels. We used a two-step method to produce highly crystalline α-MnO_2_ nanorods: (1) reduction of KMnO_4_ in the presence of natural extracts to initiate the nucleation process; and (2) a thermal treatment to enable further solid-state growth of the nuclei. Transmission electron microscopy (TEM) and field emission scanning electron microscopy (FESEM) images provided direct evidence of the morphology of the nanorods and these images were used to propose nucleation and growth mechanisms. We found that the α-MnO_2_ nanorods synthesized using natural extracts exhibit structural and magnetic properties similar to those of nanoparticles synthesized via traditional chemical routes. Furthermore, Fourier transform infrared (FTIR) shows that the particle growth of the α-MnO_2_ nanorods appears to be controlled by the presence of natural capping agents during the thermal treatment. We also evaluated the catalytic activity of the nanorods in the degradation of aqueous solutions of indigo carmine dye, highlighting the potential use of these materials to clean dye-polluted water.

## 1. Introduction

During the past decade, the synthesis of anisotropic metal oxide nanoparticles has attracted considerable attention because of their unique size- and shape-dependent properties [1,2,3]. Self-assembly processes driven by diverse interactions can generate a myriad of nanostructures by using nanocrystals as building blocks. Shape control of these structures can be achieved by tuning some of the synthesis parameters such as temperature, type of ligands, and interaction of the ligands with the precursors and/or the nanoparticles [4,5,6]. This means that the thermodynamics and kinetics of nucleation and growth can also be controlled [7,8]. Among the variety of possible shapes, nanorods are some of the most studied anisotropic materials as the combination of chemically induced growth and shape anisotropy can offer an additional level of control during self-assembly processes driven by structural and chemical selectivity [9,10].

Although many approaches have been followed for the synthesis of nanomaterials, the need to minimize toxic reagents, and to develop green synthesis processes that yield nanomaterials with similar properties to those synthesized using traditional methods remain a valid one [11]. Methods for the synthesis of nanoparticles using plant and fruit extracts are well-established in the scientific literature and several nanoparticles have been synthesized using natural extracts including gold [12,13], platinum [14], iron oxide [15,16,17], copper [18,19], palladium [20,21], zinc oxide [22], and silver [23,24,25,26,27]. Few studies have explored the synthesis of manganese oxide nanoparticles using a green synthesis route [28,29,30]. To our knowledge, there are no studies exploring the use of natural extracts for the oriented growth of highly crystalline alpha manganese dioxide (α-MnO_2_) nanorods, which is the main topic of this paper.

The synthesis of manganese oxide nanoparticles continues to be an area of active research as these particles can be used in catalysis [31], batteries [32], and supercapacitor applications [33,34]. MnO_2_ nanoparticles have been synthesized by a variety of techniques and in several shapes such as nanorods [32,33], nanowires [35], whiskers [36], and dandelion-like three dimensional structures [37]. Among the catalytic applications for manganese oxides are the degradation of dyes [38,39,40,41], water-oxidizing processes [42], and the photocatalytic oxidation of organic pollutants in waste water [43]. The most commonly used reducing agents include nitric acid (HNO_3_) [32], sodium hydroxide (NaOH) [33], hydrochloric acid (HCl) [40], and ammonium fluoride (NH_4_F) [35]. Alternative synthesis methods for manganese oxide particles include hydrothermal processes [44,45], molten salt routes [46], and microemulsion methods [47]. 

We hereby report a simple approach using natural extracts from apple peels and grape stems, to fabricate α-MnO_2_ nanorods. We found that the morphological and structural properties of the nanorods produced using these natural extracts are similar to the properties of the nanorods synthesized using traditional methods [32,33,48,49]. We also propose a mechanism to explain the formation of α-MnO_2_ nanorods. Furthermore, we tested the catalytic activity of the synthesized α-MnO_2_ nanorods on the degradation of aqueous solutions of indigo carmine dye. Finally, we found that these nanorods are very stable and suitable for repeated use. The synthesis method reported here may be easily extended to a wide variety of other metal oxide nanomaterials.

## 2. Results and Discussion

### 2.1. Materials Synthesis and Characterization

**Figure 1:**
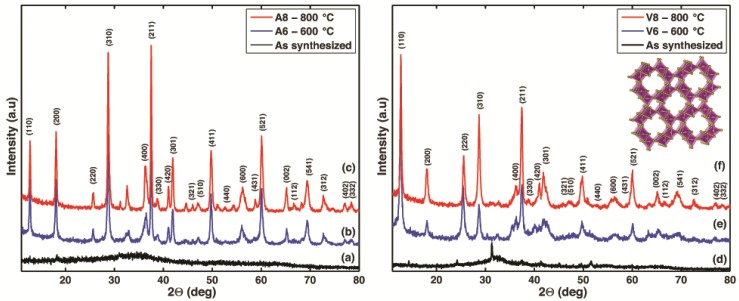
X-ray diffraction (XRD) patterns of the as-synthesized sample and alpha manganese dioxide (α-MnO) nanoparticles under different calcination temperatures (600 °C, 800 °C). (**a**–**c**) corresponds to experiments using the *Malus domestica* Cortland Apple Peels extract and (**d**–**f**) to experiments using the *Vitis vinifera* Grape stems extract.

Figure 1 shows the X-ray diffraction (XRD) patterns of samples V6 and V8, corresponding to the nanorods synthesized using the *Vitis vinifera* extracts, as well as samples A6 and A8, corresponding to the specimens synthesized with the *Malus domestica* extracts. According to the XRD patterns, the crystallinity of the synthesized products improves with further calcination at 600 °C and 800 °C, and this behavior is consistent with prior observations for the synthesis of α-MnO_2_ nanocrystals [50,51,52]. For the as-synthesized powders made with the *Malus domestica* extract (Figure 1a), few diffraction peaks can be observed. In contrast, the as-synthesized powders made with *Vitis vinifera* (Figure 1d) exhibit peaks that can be easily indexed to the potassium manganese oxide KMnO_2_ phase (Joint Committee on Powder Diffraction Standards-JCPDS, card no.: 044-1025) and MnO_2_ phase (JCPDS, card no.: 053-0633), as shown in Figure S1a. 

After thermal treatment, the crystalline structure of both samples progressively evolves towards α-MnO_2_ (JCPDS, card no.: 44-0141). XRD patterns in Figure 1b,c synthesized using the *Malus domestica* extract, exhibit a high intensity peak at 37.5°, which can be indexed as the (211) plane followed in intensity by the peaks for the planes (310) and (110). In contrast, for samples synthesized with the *Vitis vinifera* extract, Figure 1e,f, shows that the most dominant peak occurs at a plane (110) and a 2θ value of 12.5° followed by planes (211) and (310). It is important to note that regardless of the natural extract used for the synthesis, the calcinated samples at 800 °C exhibit an increase in the peak intensity and a decrease in peak broadening when compared to the same samples calcinated at 600 °C. Careful comparison of the relative intensities of the dominant diffraction peaks shows that the intensity of the peaks (110) and (220), are higher. Noting that (220) is only a short range of (110), thus, the relative intensity of the (110) peak for sample V-8 (43.2%) and for sample A-8 (100.0%) is much stronger than that shown in the standard JCPDS card (37.0%). Likewise, the relative intensity of the (220) peak for sample V-8 (35.7%) and for sample A-8 (11.62%) are 7.14 and 2.3 times the value stated in the standard JCPDS card. However, the relative intensities of the other diffraction peaks are comparable or smaller to those intensities reported in the standard JCPDS card. Thus, these results suggest a preferential growth of α-MnO_2_ in both cases [53]. It is worth noting that the calculated ratio between the intensities of the (211) and (310) diffraction peaks for samples V8 and A8 were slightly lower than that reported in the JCPDS standard (1.1 and 1.04 versus 1.2, respectively). Figure S6 shows the square tunnel structure of α-MnO_2_ with space group I 4/m, with the dominant planes highlighted by the grey shaded areas. The α-MnO_2_ structures were plotted using visualization for electronic and structural analysis (VESTA) software [54]. 

The crystallite size and the lattice parameters, calculated using the XRD patterns, are summarized in Table 1. The basic building block for most of the Mn oxide structures is the MnO_6_ octahedron, and in the α-MnO_2_ structure, these octahedra are assembled by sharing edges that form a tunnel structure (as shown in the inset of Figure 1) [55,56,57].

**Figure 2:**
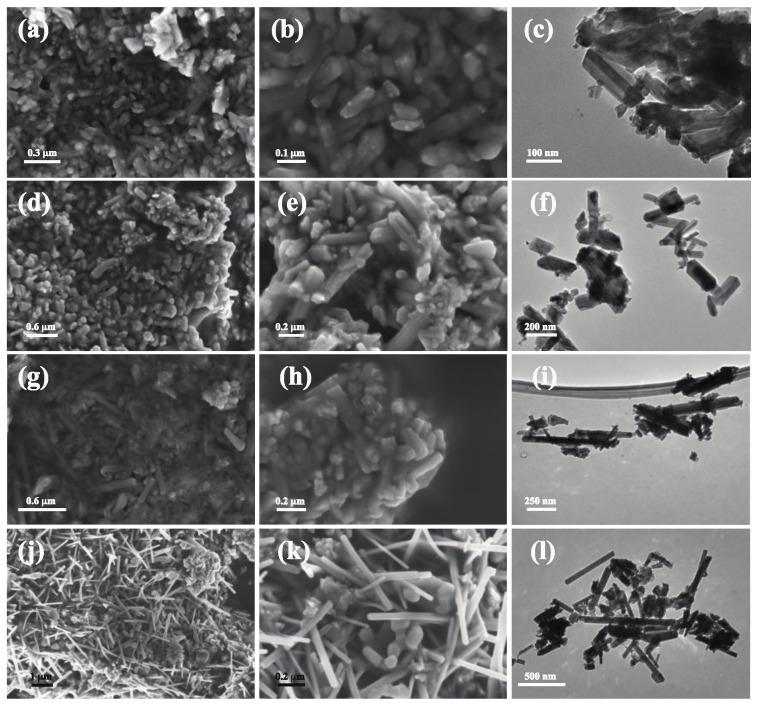
Field emission scanning electron microscopy (FESEM) and transmission electron microscopy (TEM) images of manganese oxide (α-MnO_2_) nanorods at different magnifications. (**a**–**c**) *Malus domestica* apple peel extract, calcinated at 600 °C; (**d**–**f**) *Malus domestica* apple peel extract, calcinated at 800 °C; (**g**–**i**) *Vitis vinifera* stems extract, calcination at 600 °C; (**j**–**l**) *Vitis vinifera* stems extract, calcinated at 800 °C.

Figure 2a–f shows scanning electron microscope (SEM) and TEM images of samples synthesized with the *Malus domestica* extract. These images show the presence of short and long rods that are dense and agglomerated with diameters ranging from 28 to 70 nm and lengths between 85 and 180 nm. Conversely, in Figure 2g–l, when the *Vitis vinifera* extract was used, larger nanorods were obtained and with a larger aspect ratio. The nanorods synthesized with *Vitis vinifera* do exhibit a wider particle size distribution between 150 and 800 nm in length and from 40 to 80 nm in diameter.

**Figure 3:**
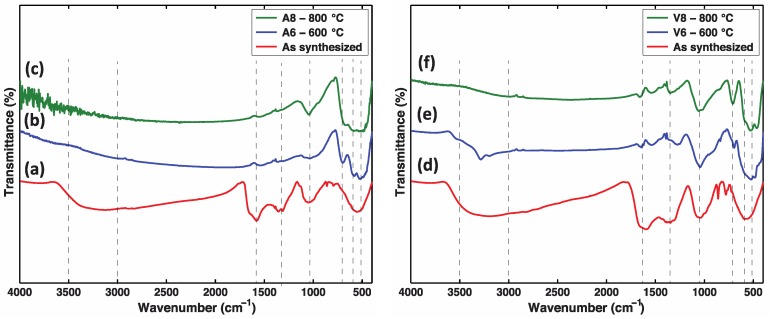
Fourier transform infrared (FTIR) spectrum of Manganese Oxide in the range from 500 to 3800 cm^−1^, KBr pellet sampling (**a**–**c**) *Vitis vinifera* stems extract; (**d**–**f**) *Malus domestica* apple peels extract.

Figure 3a–c shows the FTIR spectra corresponding to the samples prepared with the *Malus domestica* extract, while the spectra displayed in Figure 3d–f correspond to the samples prepared using the *Vitis vinifera* stems extract. The as-synthesized samples exhibited broad peaks around 3600–3000 cm^−1^, which could be assigned to the –OH stretching and H–O–H bending vibrations of bound water molecules adsorbed onto the crystalline domains [58]. The peak near 1600–1625 cm^−1^ reveals the involvement of C=O stretching vibration of acid derivatives [16]. In addition, peaks at 2900–2875 cm^−1^ and 1375–1310 cm^−1^ can be related to C–H in-plane bending and aliphatic C–O stretching modes [58]. These peaks are indicative of the presence of organic molecules that originated from the phytochemical components of the natural extracts, i.e., esters and alcohols. The spectra also exhibit peaks at 1060, 875, 850, 630, and 560 cm^−1^, which can be related to M=O stretching vibration, Mn–O–H bending, MnO_4_^−^ tetrahedral, Mn–O vibrations in octahedral environments, and Mn–O–Mn bonds, which are characteristic bands for manganese oxide [29,30,58].

Upon calcination to 800 °C, changes in the FTIR spectrum can be observed in Figure 3c,f. For example, the absorption bands assigned to the vibrations of the O–H and O–H–O groups of the absorbed water (3600–3000, 2000–1300 cm^−1^) disappear. In addition, the disappearance of the absorption bands at 1375–1355 cm^−1^ and at 850–875 cm^−1^ could be ascribed to the complete decomposition of the adsorbed phytochemicals after calcination which is confirmed by the thermal analysis presented in Figure S4. As the calcination temperature increases, the intensity of the bands at the frequencies below 750 cm^−1^ increases—these bands are ascribed to the metal-oxygen Mn–O–Mn and Mn–O bending vibrations of the [MnO_6_] octahedral in α-MnO_2_. These results are consistent with the XRD analysis reported above and are in quantitative agreement with the results reported in the literature for other MnO_2_ nanoparticles [32,36,59]. 

The thermal analysis (TG-DTG) of the as-synthesized products are shown in Figure S4. The thermogram curve and its derivative show two main stages of weight loss. The first stage occurs at about 100 °C and can be attributed to water loss (endothermic peak in the derivative curve). The second stage occurs between 300 and 375 °C and can be related to the burning of residual adsorbed phytochemicals that act as capping agents and the formation of highly crystalline α-MnO_2_. Above 600 °C, the weight loss is almost negligible. 

**Figure 4:**
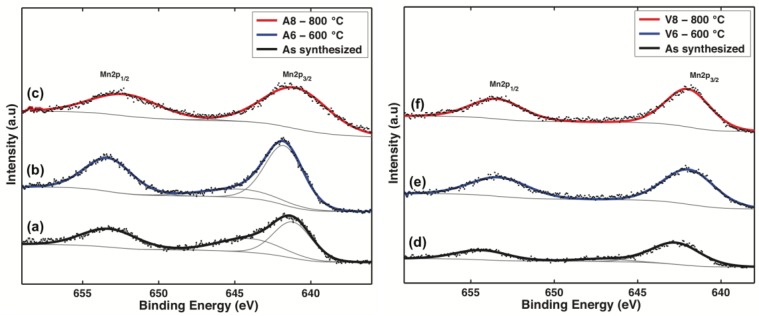
X-ray photoelectron spectroscopy (XPS) Spectra of the Manganese Oxide samples. (**a**–**c**) samples synthesized with the *Vitis vinifera* stems extract; (**d**–**f**) samples synthesized with the *Malus domestica* apple peels extract (BE values are corrected using the carbon peak at 284.6 eV as a reference).

X-ray photoelectron spectra of the six samples are shown in Figure 4. For all the samples, the binding energy of the C1s level at 284.6 eV was used as an internal reference to calibrate every spectrum to minimize charging effects. The binding energies (BE) of Mn(2p_3/2_) and Mn(2p_1/2_) are summarized in Table 2, and these values are in good accordance with those of the tetravalent manganese system MnO_2_ [60,61,62,63]. However, three samples, the as-synthesized sample with the *Malus domestica* extract, the as-synthesized sample with the *Vitis vinifera* extract, and the sample synthesized with the *Malus domestica* extract and calcinated at 600 °C (A6), all display a small but distinct shoulder at about 3 eV from the main peak. These features are also present in the calculated Mn^+4^ spectrum by Gupta and Sen [64] and Nesbitt and Barnejee [61]. This can be due to the non-stoichiometry nature of MnO_2_ hence, the occurrence of mixed valence compounds is also likely to be involved in the overall reaction [64,65]. As can be seen in Table 2, the presence of Mn^+4^ in the Mn(2p) spectrum is appreciable in supporting the findings from the XRD experiments. The spectrum O 1 s in Figure S5 reveals three types of the O atom in the as-synthesized samples. The first is assigned as a lattice oxygen Mn–O–Mn (529−530 eV) and dominates in the O1s-line, and the second can be describe as hydrated Mn–O–H manganese oxides (531–532 eV). The third corresponds to the oxygen of the absorbed water H–O–H (533 eV). After calcination, the peak assigned to the presence of lattice oxygen forming the strong Mn–O–Mn bonds in the crystalline network, progressively shifts to 529.9 eV. The intensity of the hydrated Mn–O–H peak decreases as the calcination temperature increases. All of the peak values and their evolution due to calcination are in quantitative agreement with the reported data for α-MnO_2_ [55,61,63].

**Figure 5:**
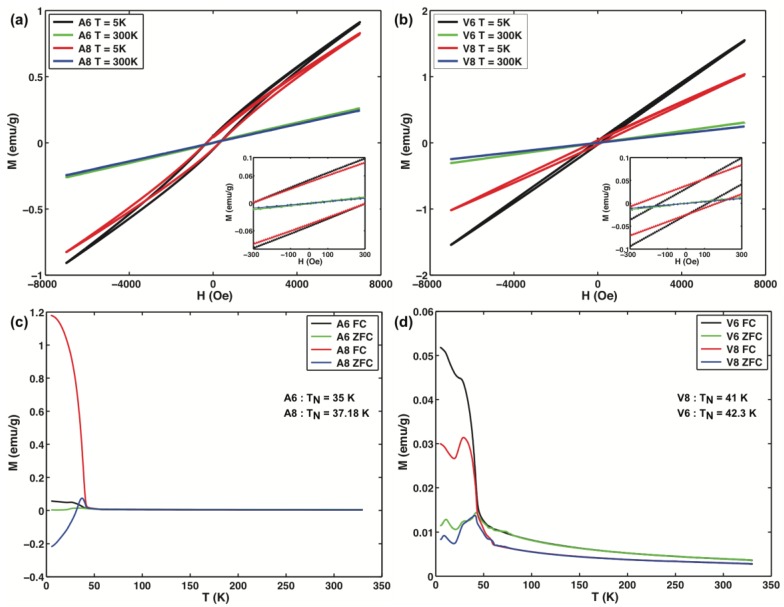
Magnetization as a function of the applied field at 5 K and 300 K for samples (**a**) A6, A8; (**b**) V6, V8; and zero-field-cooling (ZFC)/field cooling (FC) magnetization curves of the α-MnO_2_ nanorods under an applied field of 100 Oe for samples; (**c**) A6, A8; (**d**) V6, V8.

The magnetic properties of the α-MnO_2_ nanorods were measured using a vibrating sample magnetometer (VSM). Figure 5a,b shows the M–H curves for the α-MnO_2_ samples at 300 K and 5 K. The M–H curves at 300 K shows no sign of saturation even when the applied fields have a magnitude of 7 kOe. This strong linear response reveals a superparamagnetic state for the α-MnO_2_ nanorods at this temperature. However, the M–H curves at 5 K show a hysteresis loop and no sign of saturation magnetization. At this temperature, all samples exhibit exchange bias behavior that can be calculated using two fields: the exchange bias field, H_EB_, and the coercive field, H_C_. H_C_ = abs(H_C1_ − H_C2_)/2 and H_EB_ = (H_C1_ + H_C2_)/2, where H_C1_ and H_C2_ are the left and right coercive fields, respectively. Samples A6 and A8 exhibit the same coercive field and exchange bias field, 307.15 and −1.35 Oe, respectively, indicating that there is no influence of the calcination temperature on the magnetic properties of these specimens. In contrast, for samples V6 and V8 the coercive field and exchange bias field vary, 127.85 Oe and −15.55 Oe for V6 and 210.25 Oe and −44.05 Oe for V8, respectively. This variation highlights the fact that the calcination temperature, in the *Vitis vinifera* specimens, has an influence on the magnetic properties of the nanorods.

This behavior may be explained on the basis of their different preferential crystalline orientations. The exchange bias effect appears stronger in the samples synthesized with the *Malus domestica* apple peels extract than in the samples obtained with the *Vitis vinifera* grape stem extract [66]. For all samples, the magnetization of the specimens decreases with increasing calcination temperature. Figure 5c,d shows the temperature-dependent magnetization curves measured under the zero-field-cooling (ZFC) and field-cooling (FC) modes from 5 K to 300 K under 100 Oe. The observed Neel temperatures (T_N_) of the samples are lower than the ones reported for MnO_2_ bulk crystals (92 K), which is expected for the difference in the crystallite sizes [67]. In Figure 5d, a smaller peak is observed in the ZFC curve at ca. 10 K. This peak can be ascribed to the magnetic blocking of a fraction of smaller rods in the specimen [68,69]. Such magnetic behavior, including superparamagnetic responses, exchange bias field response, and the presence of multimodal blocking temperatures, hint at several interesting prospects for future studies and applications of these nanorods in magnetic nanodevices [70,71].

### 2.2. Proposed Growth Mechanism

**Figure 6:**
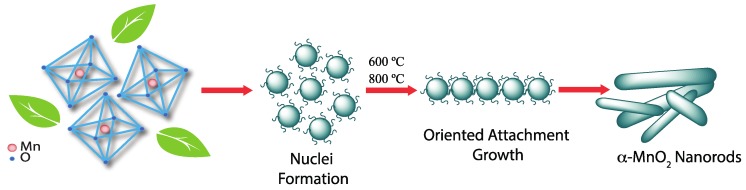
Schematic representation of the proposed nucleation and growth mechanism of α-MnO_2_ nanorods using natural extracts from *Malus domestica* and *Vitis vinifera*.

The nature of the natural extracts used in the experiments appear to have a significant morphological effect on the growth of the nanorods. Since all experimental conditions were the same, we believe that the variation of the phytochemical content in the solvent media influences the size of the α-MnO_2_ nanorods, their crystallite sizes, and their preferential crystal growth direction. A possible explanation for these results is that the phytochemicals act as reducing and stabilizer agents that control the diffusion of growth during the nucleation process [12,23]. We believe that the initial nucleation of the MnO_2_ nanorods follows the Ostwald ripening process and after nucleation, the nuclei experience a temperature-induced oriented attachment (OA) growth. In this particle-mediated crystal growth mechanism, the individual nanocrystals are integrated and fused into highly anisotropic structures with preferential crystallographic orientations [7,9]. Figure 6 shows the proposed evolution and phase transformation mechanism. 

We speculate that the natural stabilizing agents capping the nuclei particles may modulate the oriented attachment (OA) growth during the calcination processes [7,10]. The assembly appears to be driven by the presence of the phytochemicals acting as ligands and selectively binding to specific crystal faces, hence enabling the growth of the manganese oxide nanorods. The proposed mechanism appears to be supported by the XRD, thermal gravimetric analysis (TGA), and FTIR results. However, the exact role of these natural capping agents during the calcination process cannot be fully determined in this study. Figure S1b shows the HRTEM of the as-synthesized sample with *Vitis vinifera*, demonstrating that the nanorods indeed grow from the small nanoparticles. This behavior is similar to that reported by Polleux et al. [72] in which anisotropic TiO_2_ nanocrystals were synthesized by ligand-directed assembly of the nanoparticles. Similarly, Tang et al. [73] reported that the removal of excess stabilizer was the key step in the preparation of CdTe nanowires from nanoparticles. Previous studies also show morphological differences in the manganese oxide nanoparticles obtained using different solvents. For example, Yan et al. [28] synthesized Mn_3_O_4_ nanoparticles using Banana peel extract and obtained agglomerated particles with diameters from 20 to 50 nm. In contrast, Sharma et al. [30] reported the synthesis of Mn_3_O_4_ nanoparticles using the leaf extract of *Azdirachta indica* and obtained uniform spherical particles with diameters between 20 and 30 nm. 

The analysis of the chemical constituents of *Malus domestica* and *Vitis vinifera* extracts was performed via gas chromatography mass spectrometry (GC-MS). Figure S2a,b shows the mass spectra of dominant compounds in each extract and the identified compounds are shown in Table S1 and Table S2. For simplicity, we have categorized the main compounds in Table S3 into seven groups: chelating agents, alcohols, ketones, esters, carbohydrates, aldehydes, and others. The presence of chelating agents with COOH groups play a critical role in capping the nanoparticles and may control the growth during the calcination process [72,74]. Aldehydes and ketones have been reported to act as reducing agents in nanoparticle formation processes [75,76,77]. Additionally, these groups can provide colloidal stability by forming a thin layer on the surface of the nanoparticles [24,78]. Curiously, polyphenols were not detected; this may be due the nature of the solvent used in the extraction method for the GC-MS characterization. The phytochemical constituents in plants are affected by the extraction solvents shown by Abdel-Aal et al., who performed the extraction of *Spirogyra longata algea* with petroleum ether, methylene chloride, chloroform, acetone, and methanol, and found that the extraction solvent influenced the nature of the extracted molecules, especially that of alkaloid phenolics and sterols [79]. 

### 2.3. Assesing the Catalytic Activity of the Nanoparticles in Dye Degradation

**Figure 7:**
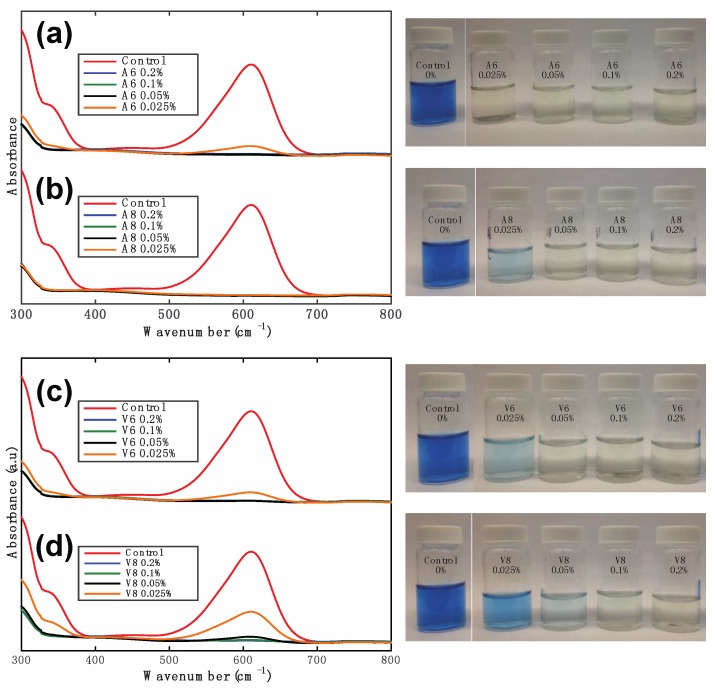
Ultraviolet-visible (UV-Vis) spectra of indigo carmine solutions after exposure to α-MnO_2_ nanorods at various concentrations. (**a**,**b**) samples synthesized with the *Malus domestica* apple peels extract; (**c**,**d**) samples synthesized with the *Vitis vinifera* grape stems extract. The right hand side shows digital photographs of the color change of the specimens.

Figure 7 shows the degradation behavior of a 20 ppm solution of indigo carmine (IC) dye in the presence of the α-MnO_2_ nanorods. The degradation of the dye solution was monitored by ultraviolet-visible (UV-Vis) spectrophotometry, using its characteristic absorption at 610 nm. For the degradation studies, we used the optimized conditions reported elsewhere [38]: briefly, the pH of the 20 ppm indigo carmine solution was adjusted to 2.5, by the addition of CH_3_COOH, and after pH adjustment, the α-MnO_2_ nanorods were added at different concentrations (0.2%, 0.1%, 0.05%, 0.025% *w/v*). The samples were vortexed for 30 s and centrifuged for 15 min at 10,000 rpm. The percentage of degradation was calculated using the following formula: %Degradation = (*A*_0_ − *A_f_*) × 100/*A*_0_, where, *A*_0_ is the initial absorbance and *A_f_* is the final absorbance at 610 nm. The UV-Vis curves in Figure 7 indicate that even at the lowest concentration α-MnO_2_-0.025% (*w/v*), there is at least 80% degradation. This demonstrates that the catalytic efficiency of these α-MnO_2_ nanorods is comparable to that of MnO_2_ nanoparticles synthesized using conventional procedures [32,33,36,41,47,80,81]. The reusability of the α-MnO_2_ nanorods was also investigated by collecting and using the same powder sample for up to four cycles. As shown in Figure S3b, the catalytic activity is not significantly altered by up to four cycles of degrading the IC dye.

## 3. Materials and Methods

### 3.1. Materials 

*Vitis vinifera* grape stems and *Malus domestica* apple peels (Cortland type), were obtained from a local market in Ithaca, NY, USA Potassium permanganate (KMnO_4_), was purchased from Sigma-Aldrich (St. Louis, MO, USA) and used as received. Milli-Q water with a conductivity <18.2 MΩ·cm at 25 °C (Milli-Q Millipore filter system, Millipore Co., Bedford, MA, USA) was used.

### 3.2. Infusion Preparation

*Vitis vinifera* grape stems and *Malus domestica* apple peels (Cortland type), were thoroughly rinsed with MilliQ water and dried in an oven at 70 °C. The dried biomass was ground into powder and stored at room temperature. Ten grams of dry biomass powder was mixed in 100 mL of MilliQ water (solid weight content of 0.1 g powder per mL) and refluxed for 1 h at 70 °C. The suspension was filtered using a cotton fabric to remove the solid particles. The suspension was centrifuged at 10,000 rpm for 10 min and filtered through a 0.2 μm filter (Millipore, Bedford, MA, USA). The pH value of the infusions ranged between 3.7 and 4.0. 

### 3.3. Nanoparticle Synthesis

Ten mL of a 0.1 M aqueous solution of KMnO_4_ was added dropwise to 40 mL of the filtered infusions at 25 °C under continuous stirring. After 2 h, a black precipitate was observed, indicating the initial formation of manganese oxide nanoparticles. The precipitate was washed several times with DI water and centrifuged at 10,000 rpm for 15 min. The resulting powders were dried in an air oven at 80 °C for 24 h, calcinated using a 30 °C/min ramp up to the target temperature (600 °C, 800 °C), and kept at this temperature for 4 h.

### 3.4. Dye Degradation Experiments

The catalytic activity of the synthesized α-MnO_2_ nanorods was assessed by dye degradation of aqueous solutions of indigo carmine (IC). Indigo carmine (5,5-indigodisulfonic acid sodium salt) is one of the oldest synthetic dyes known and is still one of the most used dyes in the textile industry [82]. The primary color-producing structure of indigoid dyes is a cross-conjugated system or H-chromophore, consisting of a single –C=C– double bond substituted by two NH donor groups and two CO acceptor groups [83]. The dye stock solution was prepared by dissolving 0.020 g of Indigo Carmine in 1000 mL of MilliQ water. Ten mL of the dye stock solution were placed in 20 mL vials, and the pH was adjusted to 2.5 using acetic acid. α-MnO_2_ powders were added at different weight/volume percent concentrations (0.2%, 0.1%, 0.05%, 0.025%) to the pH adjusted vials. The vials were vortexed for 30 s and centrifuged at 10,000 rpm for 15 min. The recovered supernatant was analyzed, immediately after centrifugation, using a Perkin–Elmer Lambda 35 UV-Vis spectrophotometer. The absorption peak was set at 610 nm. The control sample consisted of 20 ppm of indigo carmine dye, at pH 2.5, without manganese oxide. The percentage of degradation was calculated using the ratio between the characteristic UV-Vis absorption band of indigo carmine in solution at λ = 610 nm before and after contact with the α-MnO_2_ powder. 

### 3.5. Characterization

The morphology of the α-MnO_2_ powders was investigated using a field emission scanning electron microscope (FESEM) (LEO 1550) (Carl Zeiss, Oberkoehen, Germany) with an accelerating voltage of 5 kV. TEM images were collected on a FEI Tecnai T12 Spirit TEM (FEI, Hillsboro, OR, USA) with an acceleration voltage of 120 keV. Samples for FESEM and TEM imaging were drop casted onto carbon coated copper grids and left to dry at ambient conditions. The samples were further characterized using a Scintag X-ray Diffractometer (Scintag Inc., Cupertino, CA, USA) with Cu–Kα radiation (40 KV, 40 mA). The XRD patterns were recorded at room temperature with a step-size of 0.02° in the range of 10°–80°. Fourier transform infrared (FTIR) spectra of the samples were acquired using a Bruker Hyperion FTIR Spectrometer (Bruker Optics, Billerica, MA, USA) in potassium bromide (KBr) pellet transmission (KPT) mode. The KBr powders and the α-MnO_2_ powders were dried overnight in a vacuum oven prior to pellet preparation. The ratio of the sample powders to KBr was 1 mg of sample to 300 mg of KBr. X-ray photoelectron spectroscopy (XPS) measurements were performed in a surface science instruments (SSI) model SSX0100 (Surface Science Instruments, Mountain View, CA, USA) with monochromatic aluminum K-α X-rays (1486.6 eV). The analysis of peak binding energy (BE) and deconvolution was performed using the CASAXPS software (Casa Software Ltd, Teignmouth, UK), in which a Shirley background subtraction was used, as well as 50:50 Gaussian:Lorentzian line widths. All XPS measurements were corrected for charging using the C 1 s peak position at 284.8 eV. A physical properties measurement system (PPMS), equipped with a vibrating sample magnetometer (VSM), by Quantum Design (San Diego, CA, USA), was used to collect zero-field-cooled (ZFC) and field-cooled (FC) magnetization curves from 5 K to 300 K in an applied field of 100 Oe. In addition, isothermal magnetizations and hysteresis loops were collected at 300 K and 5 K for the applied fields ranging from −7 kOe to 7 kOe. UV spectrophotometry spectra were collected on a Perkin Elmer Lambda 35 spectrometer. The phytochemicals in the extracts were identified by gas chromatography-mass spectrometry (GC-MS). The GC-MS analysis was performed on an Agilent 6890 N gas chromatograph (Agilent Technologies, Santa Clara, CA, USA) equipped with an Agilent 7683 automatic liquid sampler coupled to an Agilent 5973 N mass selective detector. The extracts for phytochemical analysis were prepared using 25 mg/mL of biomass powder with high performance liquid chromatography (HPLC) grade alcohol. This mixture was stirred during 8 h, and the extracts were centrifuged and filtered through a 25-mm syringe filter. The mobile phase (Helium) was injected at a flow rate of 1.2 mL min^−1^, at a pressure of 10 psi, and with an injector temperature of 270 °C. The other parameters were adjusted as follows: the temperature of the column was initiated at 50 °C and held for 5 min, then increased to 210 °C at a rate of 15 °C min^−1^. Finally, the temperature was increased to 305 °C at a rate of 20 °C min^−1^ and held for 40 min. The samples were injected in the splitless mode and each injection was 5.0 μL. The MS detector was operated in full scan mode (35–600 amu). The retention times of each sample were compared to those reported in the national institute of standards and technology (NIST) mass spectral database. Figure S2 shows the GC-MS spectra of the extracts and a list of the constituents is provided in Tables S1 and S2. 

## 4. Conclusions

We report a facile strategy to synthesize functional α-MnO_2_ nanorods using natural extracts of apple peels and grape stems, follow by a sequential calcination process that induces an oriented attachment growth. We found evidence that the phytochemicals in the extracts act as both reducing and protecting agents, and during the calcination process enable the transformation of the stable nuclei into chemically ordered nanorods with preferential orientations. This facile synthetic strategy may provide a way to explore the formation of MnO_2_ nanostructures with different crystal phases and morphologies. The reported synthesis method, based on the separation of nucleation and the subsequent growth processes, may serve as a strategy for the further investigation of crystal growth in anisotropic nanoparticles. The results also show that the nanorods, synthesized with natural extracts exhibit interesting catalytic properties including the efficient degradation of dyes. These high-crystalline α-MnO_2_ nanorods represent a new platform for further studies of nanoscale phenomena as well as for applications such as water purification.

## Figures and Tables

**Table 1:** Crystallite size and the lattice parameters of the α-MnO_2_ nanorods produced with the *Malus domestica* apple peel extract (A6 and A8) and with the *Vitis vinifera* stems extract (V6 and V8).

Sample Name	Extract Used in the Synthesis	Calcination Temperature (°C)	Crystallite Size (Å )	a (Å)	b (Å)
A6	*Malus domestica*	600	D211: 314	9.892	2.873
A8	*Malus domestica*	800	D211: 352	9.814	2.861
V6	*Vitis vinifera*	600	D110: 243	9.922	2.854
V8	*Vitis vinifera*	800	D110: 356	9.874	2.861

**Table 2:** Mn(2p_3/2_) and Mn(2p_1/2_) spectral fitting and peak binding energy (eV) maxima

Sample	Extract	Temp (°C)	Mn(2p_3/2_) (eV)	Mn(2p_3/2_) (eV), pk 2	Mn(2p_1/2_) (eV)
As-synthesized	*Malus domestica*	--	641.19	643.99	653.26
A6	*Malus domestica*	600	641.75	644.55	653.33
A8	*Malus domestica*	800	640.99	--	652.22
As-synthesized	*Vitis vinifera*	--	642.7	647.1	654.33
V6	*Vitis vinifera*	600	641.92	--	653.28
V8	*Vitis vinifera*	800	641.97	--	653.43

## Supplementary Materials

The following are available online at http://www.mdpi.com/2079-4991/7/5/117/s1.
