# Supplementary material for: Oriented Growth of α-MnO2 Nanorods Using Natural Extracts from Grape Stems and Apple Peels
Source: Nanomaterials (Basel). 2017 May 22;7(5):117. doi: 10.3390/nano7050117 (PMC5449998; doi:10.3390/nano7050117)
Supplement: Supplementary file 1 [file nanomaterials-07-00117-s001.pdf]

Electronic Supplementary Material

Oriented growth of α-MnO_2 nanorods using natural extracts from grape stems and apple peels

Lina Sanchez-Botero [1], Adriana P. Herrera [2] and Juan P. Hinestroza [1,*]

[1] Department of Fiber Science and Apparel design, Cornell University, Ithaca, NY; lms374@cornell.edu(LSB)
[2] Department of Chemical Engineering, Universidad de Cartagena, Cartagena, Colombia; aherrerab2@unicartagena.edu.co
* Correspondence: jh433@cornell.edu; Tel.: +1-607-255-7600

This section includes: Figures S1 to S6, Tables S1 to S4.

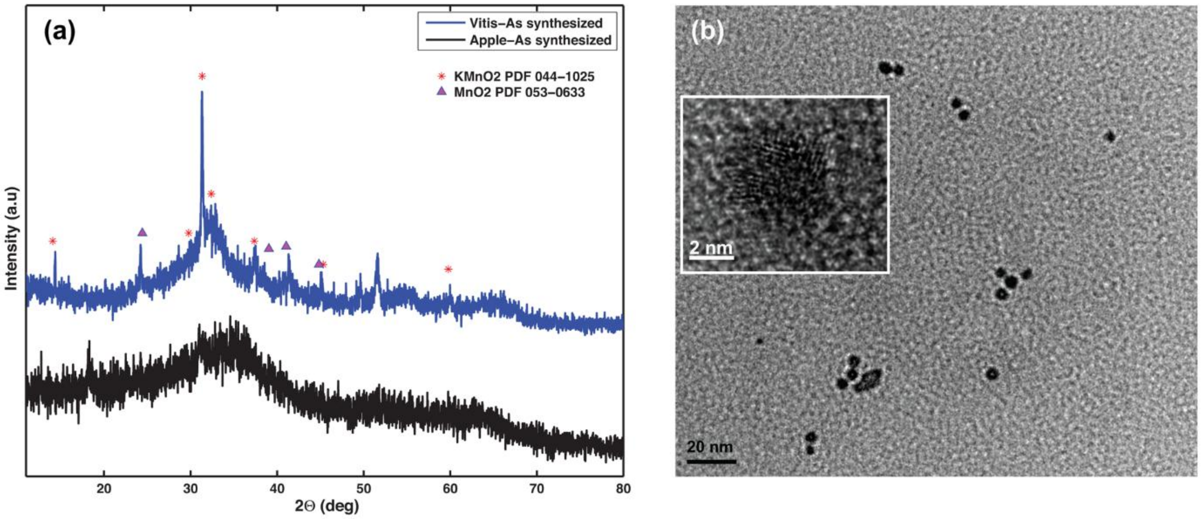

Figure S1: XRD of the (**a**) as-synthesized products using *Malus Domestica* Cortland apple peels extract and *Vitis Vinifera* stems extract; (**b**) HRTEM of the as-synthesized products using *Vitis Vinifera* stems extract.

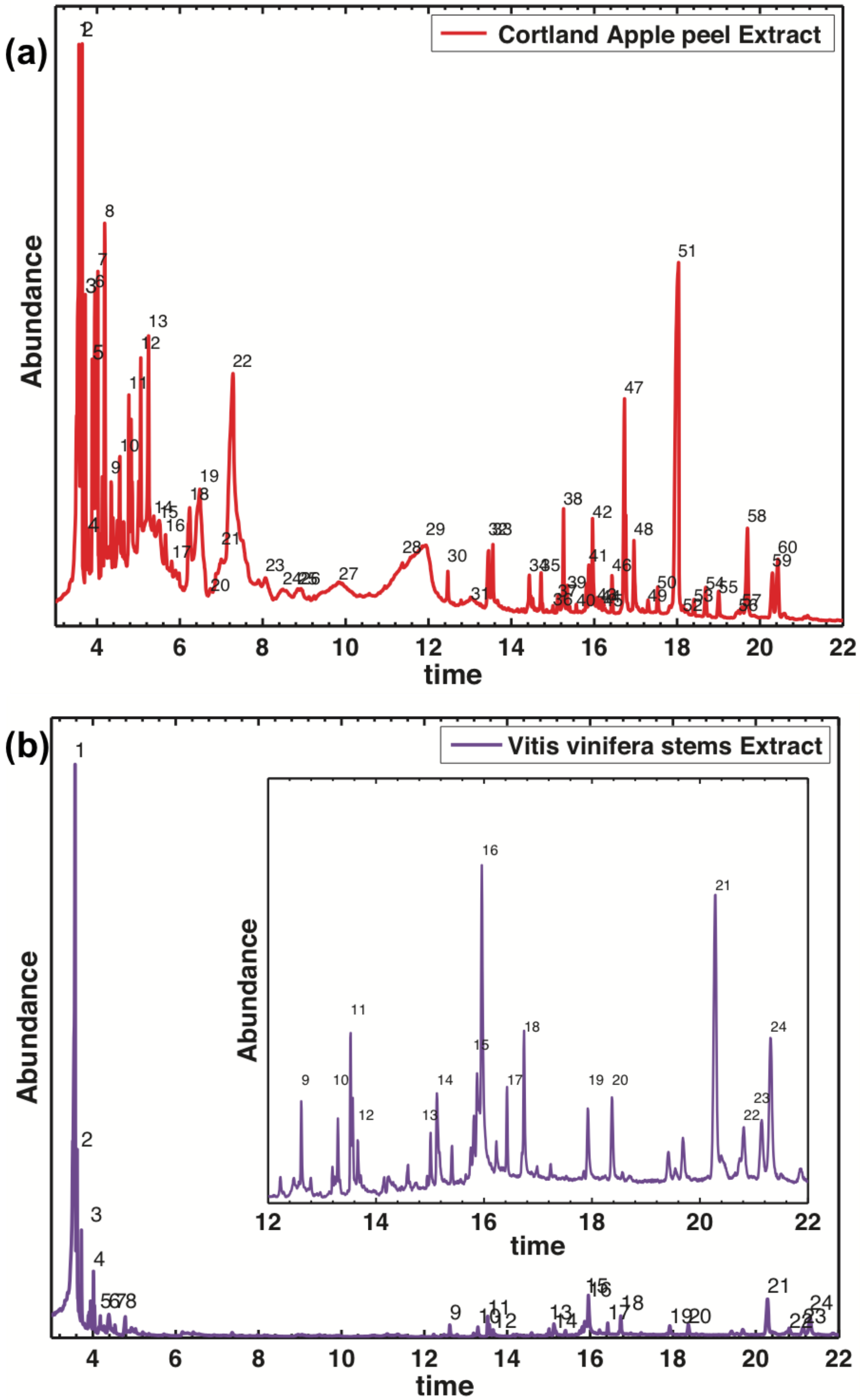

Figure S2: HPLC-MS chromatograms showing the phytochemical content in (**a**) *Malus Domestica* Cortland apple peels extract and (**b**) *Vitis Vinifera* stems extract

Table S1. GC-MS of the *Malus Domestica* Cortland Apple peel extract in Alcohol

Peak	RT	Area Pct	Library/ID	Ref	CAS	Qual
1	3.55923	6.78413	Isopropyl Alcohol	110736	000067-63-0	9
2	3.63613	4.38217	Acetic acid	109429	000064-19-7	90
3	3.72587	1.38917	2-Propanone, 1-hydroxy-	109188	000116-09-6	9
4	3.78355	0.570756	1-Propyne, 1-(methylthio)-	34894	022174-51-2	32
5	3.8989	2.26406	2-Pentanone	109359	000107-87-9	23
6	3.95017	1.73344	Propanoic acid, 2-methyl-, methyl ester	109136	000547-63-7	9
7	4.02067	2.06944	Formamide, N-methoxy-	12266	034005-41-9	47
8	4.19372	4.22977	2-Furanmethanol	108907	000098-00-0	96
9	4.34753	1.88359	2-Cyclopentene-1,4-dione	118251	000930-60-9	59
10	4.55262	3.59173	1,3-Cyclopentanedione	118435	003859-41-4	53
11	4.77052	4.16791	Ethanol, 2,2-diethoxy-	12272	000621-63-6	42
12	5.05892	3.33382	2-Pentanethiol	5934	002084-19-7	43
13	5.24477	4.49015	n-Propyl acetate	109775	000109-60-4	28
14	5.37295	1.37233	Butanoic acid, 3-hydroxy-	4897	000300-85-6	45
15	5.50113	2.60272	Butanoic acid, 3-hydroxy-	4897	000300-85-6	45
16	5.65495	1.48268	6,7-Dioxabicyclo(3,2,2)nonane	423	1000143-23-2	25
17	5.80875	0.385482	Propanal, 2,3-dihydroxy-, (S)-	5955	000497-09-6	53
18	6.23815	1.97095	Propanal, 2,3-dihydroxy-, (S)-	5955	000497-09-6	38
19	6.4817	4.5801	4H-Pyran-4-one, 2,3-dihydro-3,5-dihydroxy-6-methyl-	4836	028564-83-2	53
20	6.74447	0.219217	d-Glycero-d-galacto-heptose	6586	1000130-14-4	37
21	7.00722	1.61295	Octanal	109415	000124-13-0	38
22	7.2764	11.8662	2-Furancarboxaldehyde, 5-(hydroxymethyl)-	118397	000067-47-0	90
23	8.0711	0.752303	Silane, trichlorodocosyl-	4341	007325-84-0	38
24	8.48127	0.552728	Imidazole, 2-fluoro-5-hydroxy-1-ribofuranosyl-	4454	1000129-58-2	45
25	8.85298	0.246185	d-Mannitol, 1,4-anhydro-	110105	007726-97-8	47
26	8.92348	0.214207	d-Mannitol, 1,4-anhydro-	110105	007726-97-8	43
27	9.83997	1.69995	Methyl 2,6-anhydro-.alpha.-d-altroside	17186	1000130-02-0	38
28	11.3717	2.32388	Ethanol, 2-(2-aminoethoxy)-	771	000929-06-6	50
29	11.9357	7.0934	Oxirane, 2,3-dimethyl-	109412	003266-23-7	43
30	12.4676	0.308145	n-Hexadecanoic acid	109984	000057-10-3	96
31	13.0252	0.502538	Sorbitol	114905	000050-70-4	72
32	13.4482	0.667802	9,12-Octadecadienoic acid (Z,Z)-	113793	000060-33-3	95
33	13.5571	0.70974	Octadecanoic acid	109986	000057-11-4	98
34	14.4352	0.510624	9,17-Octadecadienal, (Z)-	21389	056554-35-9	94
35	14.7172	0.316285	9,12-Octadecadienal	2320	026537-70-2	86
36	15.012	0.0583331	Tritetracontane	17076	007098-21-7	43
37	15.1273	0.147779	Hexadecanoic acid, 2,3-dihydroxypropyl ester	5500	000542-44-9	86
38	15.2619	0.694506	2-Methyl-Z,Z-3,13-octadecadienol	12982	1000130-90-5	94

39	15.3516	0.230629	Decanoic acid, hexyl ester	4129	010448-26-7	47
40	15.5695	0.0534772	Oxirane, tetradecyl-	108584	007320-37-8	72
41	15.8707	0.644844	9,17-Octadecadienal, (Z)-	21389	056554-35-9	97
42	15.9669	0.699975	11-Undecanolide	14557	1000132-46-4	76
43	16.0822	0.151466	Oleic Acid	108586	000112-80-1	64
44	16.172	0.12148	Decanoic acid, hexyl ester	4129	010448-26-7	53
45	16.2296	0.144815	Oxirane, [(hexadecyloxy)methyl]-	16342	015965-99-8	58
46	16.4347	0.243017	Squalene	114263	007683-64-9	80
47	16.7296	2.2583	Hexatriacontane	112334	000630-06-8	91
48	16.9603	0.761368	9,17-Octadecadienal, (Z)-	21389	056554-35-9	93
49	17.2999	0.152244	2-Octadecyl-propane-1,3-diol	5166	005337-61-1	58
50	17.5307	0.224429	Oxirane, tetradecyl-	1807	007320-37-8	87
51	18.0241	7.09394	3-Eicosene, (E)-	16986	074685-33-9	58
52	18.1459	0.0520822	1-Dotriacontanol	16786	006624-79-9	20
53	18.4087	0.11347	D,.alpha.-Tocopherol	106155	1000128-08-6	96
54	18.6907	0.264057	2,6,10-Dodecatrien-1-ol, 3,7,11-trimethyl-, acetate, (E,E)-	114194	004128-17-0	76
55	19.0047	0.275587	Octadecanal	4350	000638-66-4	91
56	19.4854	0.129441	Acetic acid, 6-hydroxymethyl-cyclodecyl ester	6823	1000190-93-2	27
57	19.5623	0.151766	17-Pentatriacontene	5525	006971-40-0	76
58	19.6969	1.17486	Cyclohexane, 1,2-dimethyl-3-pentyl-4-propyl-	42165	062376-17-4	47
59	20.2993	0.545766	.gamma.-Sitosterol	109486	000083-47-6	99
60	20.4339	0.731765	2,6,10-Dodecatrien-1-ol, 3,7,11-trimethyl-, acetate, (E,E)-	114194	004128-17-0	87

Table S2. GC-MS of the *Vitis Vinifera* (grape) stems extract in Alcohol

Peak	RT	Area Pct	Library/ID	Ref	CAS	Qual
1	3.56545	65.0887	Isopropyl Alcohol	110736	000067-63-0	9
2	3.72567	3.3399	2-Propanol, 1-amino-	710	000078-96-6	9
3	3.94998	1.96949	Monoethanolamine	108158	000141-43-5	9
4	4.02048	2.57911	Formamide, N-methoxy-	12266	034005-41-9	59
5	4.1807	1.77931	1,3-Butanediol	109434	000107-88-0	28
6	4.38578	2.45411	Oxirane, ethyl-	3255	000106-88-7	12
7	4.5396	0.579408	4,5-Dihydro-2-methylimidazole-4-one	3438	1000128-69-3	52
8	4.78313	0.951469	Pentanoic acid	113425	000109-52-4	72
9	12.6212	0.634592	Hexadecanoic acid, ethyl ester	35861	000628-97-7	93
10	13.2942	0.859799	Phytol	114545	000150-86-7	90
11	13.5313	1.48941	2-Chloroethyl linoleate	21410	025525-76-2	93
12	13.6659	0.540413	Nonadecanoic acid, ethyl ester	35652	018281-04-4	86
13	15.0117	0.366863	Octadecane, 1-chloro-	109594	003386-33-2	87
14	15.1335	1.03709	15-Hydroxypentadecanoic acid	14560	004617-33-8	53
15	15.877	1.92916	9,12-Octadecadienoic acid (Z,Z)-, 2-hydroxy-1-(hydroxymethyl)ethyl ester	21417	003443-82-1	74
16	15.9667	3.20876	Cyclohexanone, 4-(1,1-dimethylethyl)-	112682	000098-53-3	38
17	16.4281	0.488607	Squalene	114263	007683-64-9	86
18	16.7422	1.20815	1-Docosene	111438	001599-67-3	93
19	17.9278	0.72115	1-Dotriacontanol	16786	006624-79-9	90
20	18.3764	0.723047	D,.alpha.-Tocopherol	106155	1000128-08-6	96
21	20.2863	4.23136	.gamma.-Sitosterol	109486	000083-47-6	99
22	20.8118	0.918306	2(1H)Naphthalenone, 3,5,6,7,8,8a-hexahydro-4,8a-dimethyl-6-(1-methylethenyl)-	87110	086941-63-1	62
23	21.1451	0.814287	2,6,10-Dodecatrien-1-ol, 3,7,11-trimethyl-, (Z,E)-	114132	003790-71-4	47
24	21.3117	2.08747	1H-Cycloprop[e]azulene, decahydro-1,1,7-trimethyl-4-methylene-, (1a.a	3099	072747-25-2	35

Table S3. Main Phytochemical compounds for *Malus domestica* (Apple peel) and *Vitis Vinifera* (grape stems) extracts in Alcohol

***Malus domestica* (Apple peel)**	***Vitis Vinifera* (grape stems)**
**Chelating agents**	**Chelating agents**
Acetic acid<br>Propanoic acid<br>Butanoic acid<br>Octadecanoic acid<br>Oleic Acid	Pentanoic acid<br>15-Hydroxypentadecanoic acid
**Alcohol**	**Alcohol**
Isopropyl alcohol<br>2-Furanmethanol<br>1-Dotriacontanol	Isopropyl alcohol<br>1-amino-2-Propanol<br>Ethanolamine<br>1,3- butanediol<br>Phytol<br>Dotriacontanol<br>3,7,11-trimethyl-2,6,10-dodecatrienol
**Ketones**	**Ketones**
Hydroxyacetone<br>2-Pentanone<br>1,3-Cyclopentanedione<br>Oxacyclododecan-2-one	Cyclohexanone
**Ester**	**Ester**
n-Propyl acetate<br>hexyl decanoate<br>(E,E)-Farnesyl acetate	Ethyl palmitate<br>2-chloroethyl ester<br>N-Nonadecanoic acid ethyl ester
**Carbohydrates / sugars**	**Carbohydrates / sugars**
1,4-anhydro-d-Mannitol<br>Sorbitol	
**Aldehydes**	**Aldehydes**
2,3-dihydroxy Propanal<br>Octanal<br>Aldoheptose (d-Glycero-d-galacto-heptose)<br>9,12-Octadecadienal<br>Octadecanal	
**Others**	**Others**
Vitamin E	Vitamin E<br>N-Methylformamide

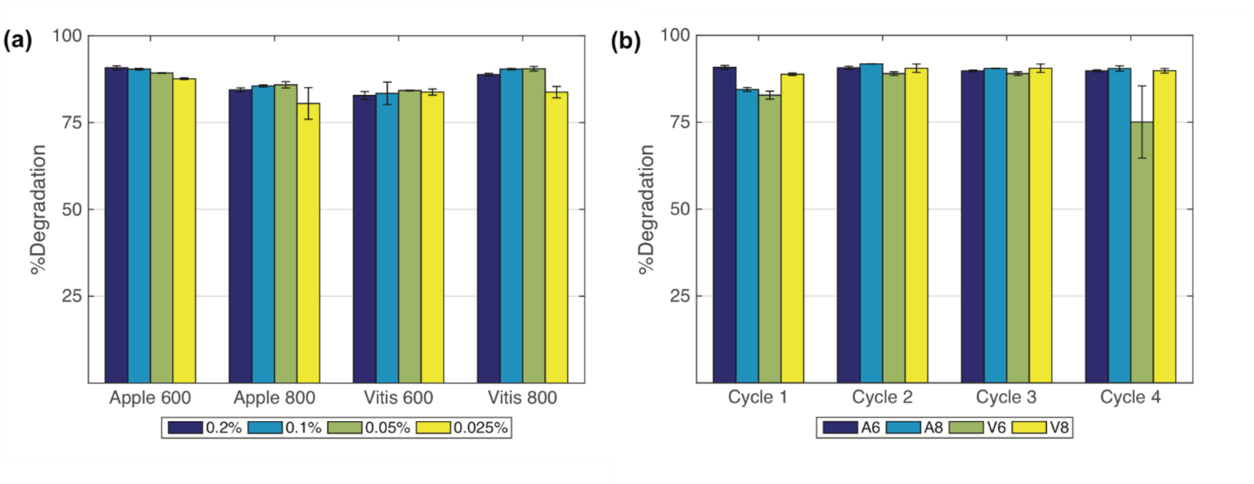

Figure S3: Dye degradation as a function of mass percent concentration of (**a**) α-MnO_2 products of Apple 600, Apple 800, Vitis 600 and Vitis 800 and (**b**) Reusability cycles comparison of samples Apple 600, Apple 800, Vitis 600 and Vitis 800 with 0.2% concentration. All samples had pH adjusted to 2.5.

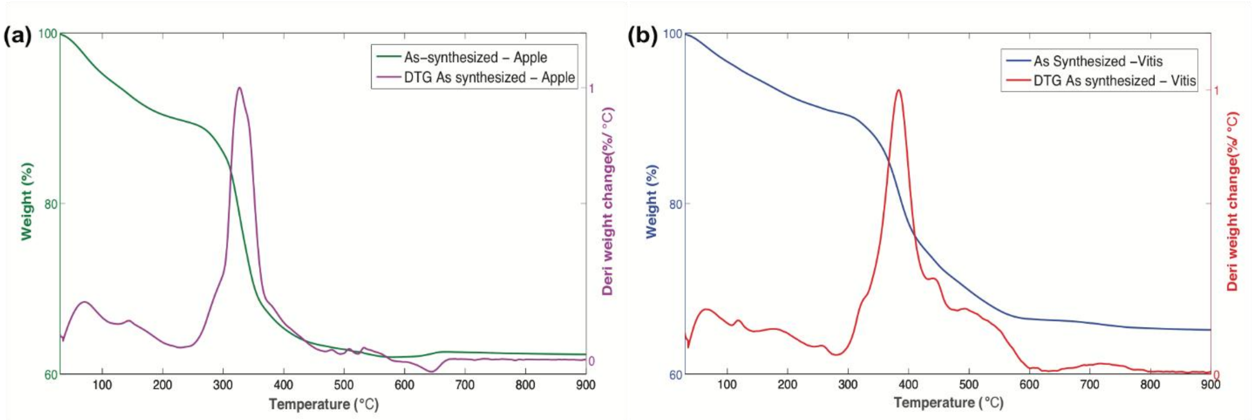

Figure S4: TGA of the as-synthesized NPs using, (**a**) *Malus domestica* apple peels extract and (**b**) *Vitis vinifera* grape stems extract

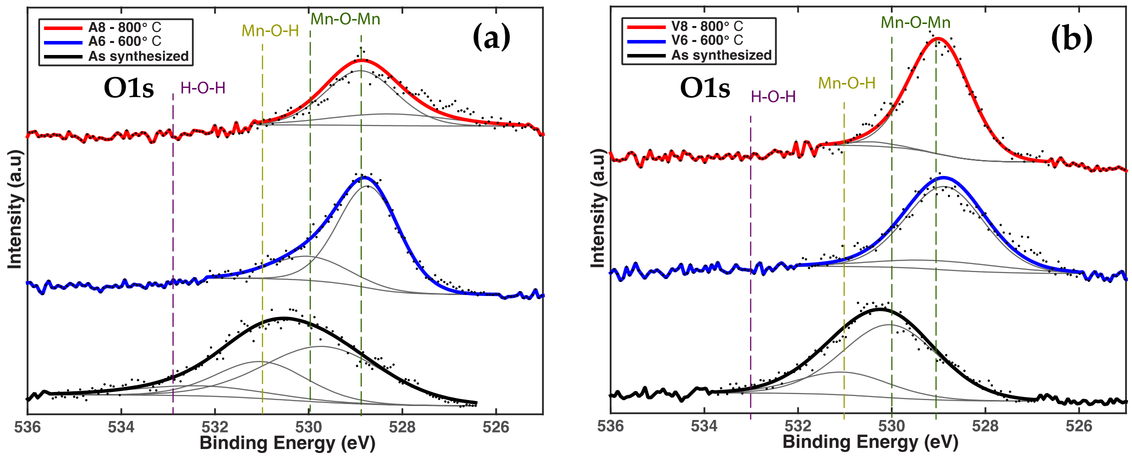

Figure S5: O 1s XPS spectra of the α-MnO_2 nanorods using, (**a**) *Malus domestica* apple peels extract and (**b**) *Vitis vinifera* grape stems extract

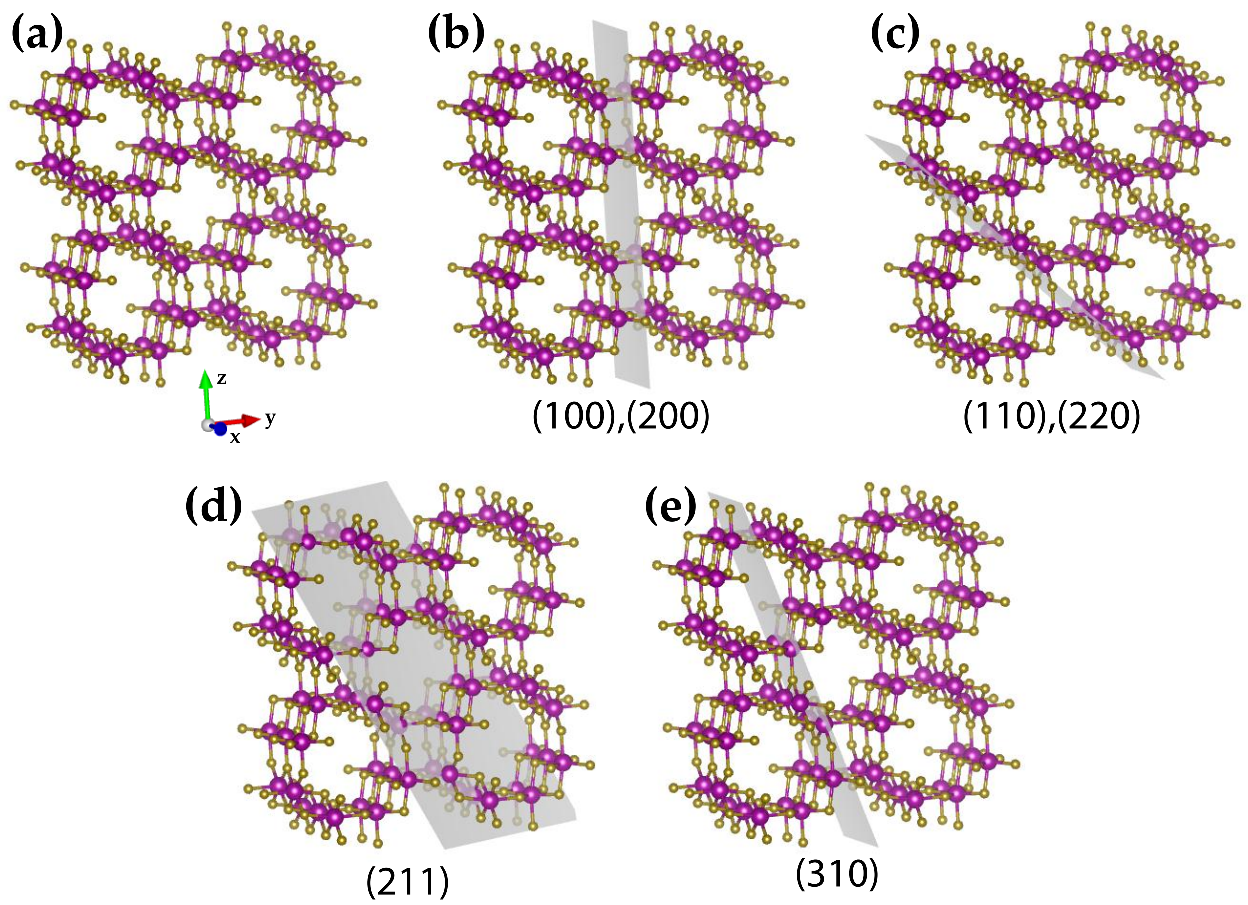

Figure S6: (**a**) Square tunnel structure of *α*-MnO2 with the space group *I*4/*m*. The crystal planes of interest are highlighted by the grey shaded area (**b**) (100), (200) (**c**) (110),(220) (**d**) (211) (**e**) (310)
